# Evaluation of response to conventional chemotherapy and radiotherapy by perfusion computed tomography in non-small cell lung cancer (NSCLC)

**DOI:** 10.1186/s41747-019-0101-x

**Published:** 2019-06-13

**Authors:** Carmen Trinidad López, Javier De La Fuente Aguado, Roque Oca Pernas, Carlos Delgado Sánchez-Gracián, Eloisa Santos Armentia, Antonio Vaamonde Liste, Raquel Prada González, Miguel Souto Bayarri

**Affiliations:** 1 Department of Radiology, POVISA Hospital, 5 Salamanca st, 36208 Vigo, Pontevedra Spain; 2 Department of Internal Medicine, POVISA Hospital, Vigo, Spain; 3 Department of Radiology, Osatek, Urduliz Hospital, Vizcaya, Spain; 4 Department of Statistics and Operational Research, Faculty of Economic and Business Sciences, Vigo University Spain, Vigo, Spain; 5 Department of Radiology, Complexo Hospitalario Universitario de Santiago de Compostela, Santiago de Compostela, Spain

**Keywords:** Carcinoma (non-small cell lung), Lung neoplasms, Perfusion imaging, Response evaluation criteria in solid tumours (RECIST), Tomography (x-ray, computed)

## Abstract

**Background:**

To evaluate changes in perfusion computed tomography (PCT) parameters induced by treatment with conventional chemotherapy (CCT) alone or with CCT and radiation therapy (RT) in patients with non-small cell lung cancer (NSCLC) and to determine whether these changes correlate with response as defined by the *response evaluation criteria in solid tumours* version 1.1 (RECIST-1.1).

**Methods:**

Fifty-three patients with a histological diagnosis of NSCLC prospectively underwent PCT of the whole tumour, before/after CCT or before/after CCT and RT. Blood flow (BF), blood volume (BV), permeability (PMB), and mean transit time (MTT) were compared before and after treatment and with the response as defined by RECIST-1.1. The relationship between changes in the perfusion parameters and in tumour size was also evaluated.

**Results:**

PCT parameters decreased after treatment, significantly for BV (*p* = 0.002) and MTT (*p* = 0.027). The 30 patients with partial response had a significant decrease of 21% for BV (*p* = 0.006) and 17% for MTT (*p* = 0.031). A non-significant decrease in all perfusion parameters was found in patients with stable disease (*p* > 0.137). In patients with progressive disease, MTT decreased by 10% (*p* = 0.465) and the other parameters did not significantly vary (*p* > 0.809). No significant correlation was found between changes in size and PCT parameters (*p* > 0.145).

**Conclusions:**

Treatment of NSCLC with platinum derivatives, with or without RT, induces changes in PCT parameters. Partial response is associated with a significant decrease in BV and MTT, attributable to the effect of the treatment on tumour vascularisation.

## Keypoints

- Perfusion parameters at computed tomography were compared before and after treatment of non-small cell lung cancer.
- Treatment-induced significant changes in perfusion parameters.
- Partial response was associated with a significant decrease in blood volume and mean transit time.
- Changes in perfusion parameters were independent of changes in tumour size.

## Background

Lung cancer is a serious health problem because it represents 13–20% of cancer diagnoses and is the most common cause of death from malignancy worldwide, with a 5-year survival rate of only 15–20% [1].

Until recently, the first line of treatment in advanced lung tumours was platinum derivatives, associated or not with radiation therapy (RT) on the tumour [2, 3]. Recently, several genomic mutations have been identified, such as those regarding the endothelial growth factor receptor and anaplastic lymphoma kinase that are associated with response to tyrosine kinase inhibitors and crizotinib, respectively, with a marked improvement in response rates and survival [4, 5].

In this setting, the assessment of response to treatment based on tumour size using the response evaluation criteria in solid tumours version 1.1 (RECIST-1.1) guidelines [6] presents some disadvantages, such as disregarding any asymmetric growth of the tumour that does not involve changes in the maximum diameter or the presence of cavitation, necrosis, or haemorrhage that increase the size of the tumour. Therefore, the development of new methods and imaging techniques to assess treatment response using parameters other than size is necessary [7–9].

As happened with targeted therapies, both conventional chemotherapy (CCT) and RT decrease tumour vascularisation [10]. It is known that CCT significantly decreases perfusion parameters in different tumours of the liver, oesophagus, and lungs due to the loss of the angiogenic effect of cytokines after cellular death [11, 12]; RT causes a reduction in the perfusion values because it acts on the vessels, causing microvascular damage [10]. Therefore, when changes in tumour vascularisation are evaluated, we can detect the effects of the treatment more reliably and earlier, thus avoiding prolonged use and toxicity in patients who do not respond, in addition to decreasing economic costs. Perfusion computed tomography (PCT) is an imaging tool that has attracted increasing attention because the parameters obtained can be an indirect reflection of the vascularisation and vascular physiology of the tumour [8–21], and it can be easily incorporated into routine monitoring protocols for lung cancer [22]**.**

As far as we know, there are only few published studies that evaluate the changes in perfusion parameters before and after treatment in patients with non-small cell lung cancer (NSCLC) [23–27]. Three of these studies include different combinations of antiangiogenic drugs [24–26] and two conventional CCT and/or RT protocols [23, 27]. The results show that PCT parameters are useful to detect early changes caused by antiangiogenic therapy but not by treatment with platinum derivatives.

The objective of our study was to evaluate the changes in PCT parameters induced by cytotoxic CCT and RT and to determine whether these changes can serve to monitor the response to treatment.

## Methods

The authors had full control of all the data and information presented in this manuscript. Written informed consent was obtained from all the patients involved in the study, and the entire study protocol was approved by the Ethics Committee.

### Patients

**Fig. 1:**
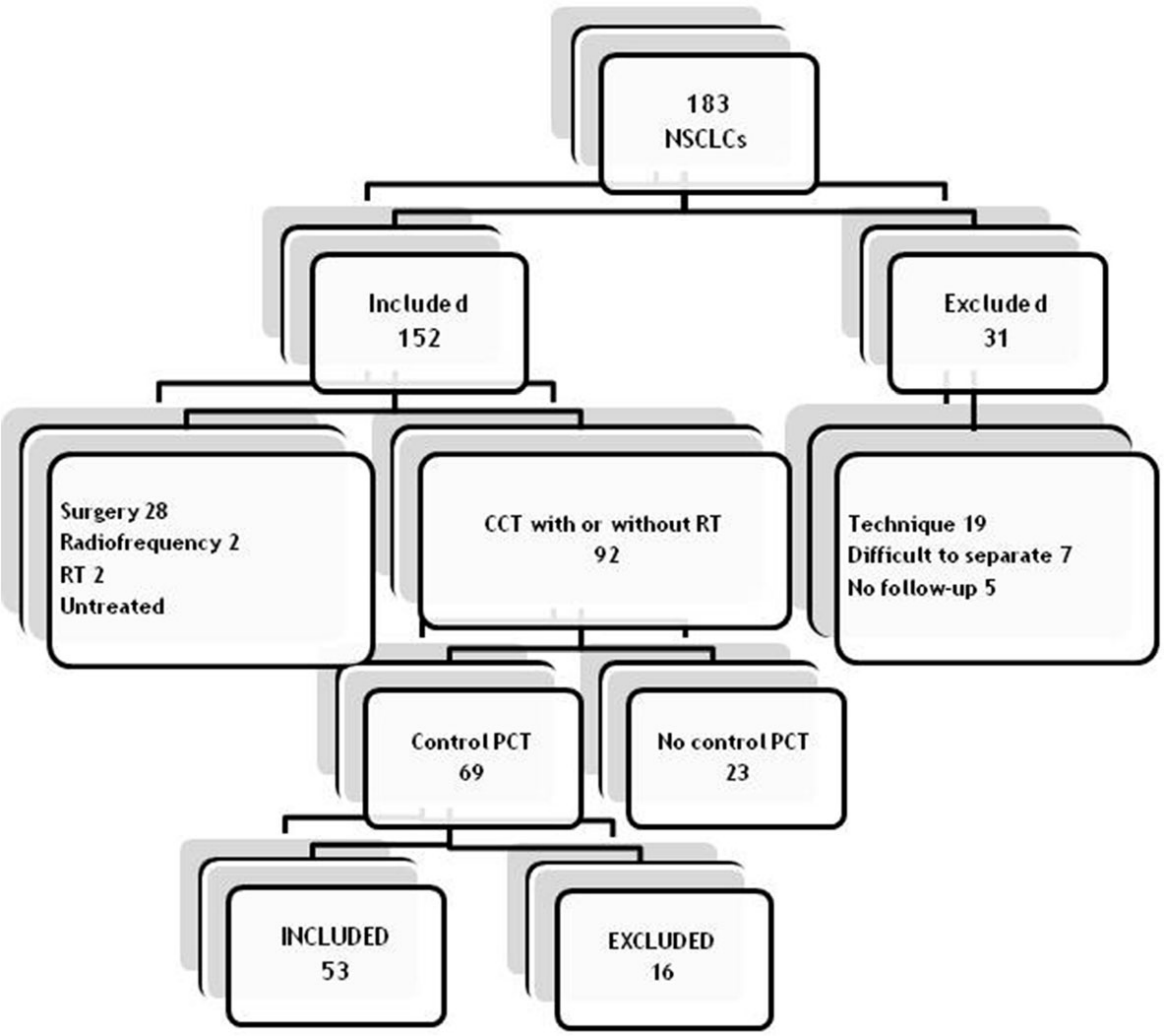
Study patients flow chart. *NSCLC* Non-small-celllung cancer, *PCT* Perfusion computed tomography, *CCT* Conventional chemotherapy, *RT* Radiation therapy

Between January 2010 and December 2015, patients with a histological diagnosis of lung cancer were evaluated by a multidisciplinary lung tumour committee and selected by a radiologist with more than 10 years of experience in chest imaging for CTP performance. Figure 1 shows a flow chart of patients included in this study.

A total of 183 patients with a histological diagnosis of NSCLC were prospectively enrolled in our study. All of them underwent PCT before receiving any treatment (baseline study). A total of 53 patients who received treatment with cytotoxic CCT or concomitant CCT and RT underwent a second PCT study following the treatment (control study).

Inclusion criteria were histological diagnosis of NSCLC; absence of previous oncological treatment for this tumour; maximum tumour diameter larger than 2 cm; and first-line treatment with cytotoxic CCT, with or without associated RT. Exclusion criteria were PCT studies of poor technical quality because of movement artefacts, x-ray beam hardening, noise, or inadequate contrast enhancement; tumours difficult to separate from other lesions such as atelectasis, pneumonitis, or lymphangitis; patients who did not continue follow-up at our hospital; complete response or lesion less than 2 cm in diameter on the first PCT control after treatment; and presence of respiratory artefacts that could not be corrected by automatic movement correction algorithms.

The baseline study was performed taking into account that iodinated contrast had not been administered in the previous 24 h. The control study was conducted during the same examination as the first standard computed tomography control after treatment with CCT, performing the PCT first and subsequently the study of the entire chest. In patients who received RT, the exam was performed 2 weeks after the end of therapy to avoid inflammatory changes that could influence perfusion parameters.

A total of 53 patients participated in this study, including 42 men (79%) and 11 women (20%), with an age of 62.4 ± 9.9 years (mean ± standard deviation; range 35–79 years). The mean dose length product (DLP) for PCT was 463.8 ± 123.3 mGy cm (mean ± standard deviation; range 273–713) and the mean effective radiation dose was 6.40 ± 1.72 mSv (mean ± standard deviation; range 3.82–9.82).

A total of 16 patients were excluded: 6 patients because of poor technical quality (4 with respiratory artefacts; 2 patients with x-ray beam hardening artefacts due to tumour proximity to the superior vena cava and the bone for a tumour located in the pulmonary apex), 7 patients because of the absence of an identifiable tumour or an unmeasurable lesion in the second PCT study, 1 patient due to RT in the previous 2 weeks, and 2 patients in whom the entire tumour volume had not been included in the second study.

The electronic clinical histories were reviewed, and the following data were collected: age, sex, tumour histology, radiological stage at the time of diagnosis according to the TNM classification, type of CCT, association or non-association with concomitant RT, time elapsed in days since the basal PCT study was performed until the first day of CCT, time elapsed in days from the start of the treatment until the second PCT control, and volume and maximum diameter of the tumour in the axial plane before and after treatment.

Two radiologists with 14- and 12-year experience in chest radiology evaluated the first PCT control and classified the patient cases as complete response (CR), partial response (PR), stable disease (SD), or progressive disease (PD) using the RECIST-1.1 criteria [6], applied only to the lesion on which the PCT studies were performed. When there was disagreement, the two readers reached an agreement by consensus.

### Patient preparation and PCT technique

Prior to the exam, the patient was trained to maintain apnoea throughout the study. When the patient was not able to breath-hold, she/he was trained to perform shallow respiration.

A dual-source equipment with 128 rows of detectors (Flash Definition®; Siemens, Forchheim, Germany) was used. Once the topogram was performed, a radiologist planned the study field, including the entire lesion along the *z*-axis. When the lesion was not clearly identified in the topogram, an unenhanced scan was performed to locate it.

Fifty mL of iodinated contrast was injected (Iopromide 300, Ultravist® Bayer Healthcare; Berlin, Germany) at 5 mL/s, followed by 50 mL of saline at the same rate.

The PCT acquisition was initiated 2 s after the injection of the contrast commenced, using the following parameters: 80 kVp and 90 mAs; 32 × 1.2-mm detector configuration; 0.33 s tube rotation time; 3 or 5 mm reconstructed image thickness, according to the tumour size; and B20f reconstruction kernel. The total time of the PCT study was always 45 s. The time interval between scans was 1.5 or 1 s, depending on the tumour size along the *z*-axis, which resulted in 30 to 45 scans in each tumour. The total length of the studies along the *z*-axis ranged from 4 to 15 cm.

### Post-processing and image analysis

The data were transferred to a workstation (Multi-Modality Workplace®, Siemens, Forchheim, Germany) and processed using the Volume Perfusion Computed Tomography (VPCT) Body program. The PCT studies were post-processed and analysed by a senior chest radiologist with 14 years of experience who has received specific training in lung cancer perfusion post-processing, without knowledge of the results of the first standard computed tomography control after treatment with CCT.

First, the automatic motion and noise correction algorithms included in the VPCT Body software were applied. An arterial density-to-time curve was obtained by placing a region of interest in the thoracic aorta, where the unenhanced reference image was selected. The tumour volume was selected via manual segmentation, drawing the contours of the lesion in the axial, coronal, and sagittal planes, using a cut-off threshold of -50–150 UH, which permitted automatically excluding the normal pulmonary parenchyma, non-tumour vascular structures, and calcium, from the segmented volume.

The following perfusion parameters were calculated using a deconvolution model: BF, in mL/100 mL/min; BV, in mL/100 mL; PMB, in mL/100 mL/min, and MTT, in seconds. Each parameter was represented as a colour on parametric maps; numerical values were given as mean and standard deviation.

### Statistical analysis

Quantile comparison graphs were used to evaluate if variables followed a normal distribution. All data were normally distributed except BF that was near-normally distributed.

The means of perfusion parameters between responders and no responders as well as between adenocarcinomas and epidermoid carcinomas were compared in baseline study using *t* test for independent samples. The means and standard deviations of the perfusion parameters were calculated for all of the patients in the baseline study and in the control study and were compared to one another using the *t* test for paired samples. The perfusion parameters were compared before and after the treatment, at the different response levels according to RECIST-1.1, for all histological subtypes, and in epidermoids and adenocarcinomas separately, using the *t* test for paired samples. The Pearson correlation coefficient was used to evaluate whether there was a relationship between changes in the perfusion parameters and changes in tumour volume or size.

The programs R (R Foundation for Statistical Computing, 2014, V 3.1.0) and SPSS® (IBM® SPSS® Statistics 20.0.0) were used for the statistical analysis. The level of statistical significance adopted was 0.05.

## Results

In all cases included, the kinetic model obtained after post-processing and imaging analysis was reliable. Patient and tumour characteristics are reported in Table 1.

**Table 1:** Patients and tumours characteristics (*n* = 53)

Smoking	48 (90.6)
Family history of lung cancer	15 (28.3)
Personal history of cancer	3 (5.7)
Comorbidities	17 (32.1)
Presentation	
Incidental finding	10 (18.9)
Solitary pulmonary nodule	1 (1.9)
Cough	50 (40.3)
Dyspnoea	18 (34.0)
Pain	13 (24.5)
Haemoptysis	12 (22.6)
Constitutional syndrome	27 (50.9)
Histology	
Adenocarcinoma	28 (52.8)
Epidermoid	16 (30.2)
Undifferentiated	5 (9.4)
Large cell	3 (5.7)
Neuroendocrine	1 (1.9)
Treatment	
CCT with platinum	46 (86.8)
CCT and RT	7 (13.2)
Stage	
IIB	3 (5.7)
IIIA	7 (13.2)
IIIB	11 (20.8)
IV	32 (60.4)
RECIST-1.1 diameter (mm)	59 ± 22 (21–115)
Volume (cc)	107 ± 137 (1–605)
Time from baseline PCT to CCT start (days) 29.37 ± 15.8 (8–82)	
Time from CCT start to control PCT (days) 63.9 ± 28.1 (42–138)	

Data are given as absolute frequencies with percentages in parentheses or means ± standard deviations with ranges in parentheses. *CCT* Conventional chemotherapy, *PCT* Perfusion computed tomography, *RECIST* Response evaluation criteria in solid tumours, *RT* Radiation therapy

In the first PCT control after the treatment, 30 patients were classified as PR, 20 as SD, and 3 as PD. There were no significant differences between the response groups in terms of age (*p* = 0.462), tumour size (*p* = 0.559), or tumour volume (*p* = 0.441), nor the elapsed time between the baseline perfusion study and the initiation of treatment with chemotherapy (*p = 0.803*). In the patients with a PR to treatment, the time elapsed between the initiation of CCT treatment and the PCT control study was significantly longer than in the non-responders (*p* = 0.002)*.*

There were no significant differences in perfusion parameters in the baseline study between responders and non-responders: BF 162.00 *versus* 225.87, *p* = 0.142; BV 8.13 *versus* 9.04, *p* = 0.42; PMB 15.48 *versus* 17.85, *p* = 0.340; and TTM 7.05 *versus* 6.58, *p* = 0.64. The same was for the response levels according to RECIST-1.1: BF, *p* = 0.208; BV, *p* = 0.494; PMB *p* = 0.587; and TTM *p* = 0.727.

The mean values of all perfusion parameters decreased in the control perfusion study after the treatment, and the difference was significant for BV and MTT (Table 2). The 46 patients treated only with CCT presented a decrease in all perfusion values after treatment, significant for BV (*p* = 0.030) and MTT (*p* = 0.023).

**Table 2:** Values of perfusion parameters, RECIST-1.1 diameter, and tumour volume in baseline and control perfusion computed tomography (*n* = 53)

	Baseline	Control	*p* value
Blood flow (mL/100 mL/min)	189.7 ± 141.8	170.9 ± 100.7	0.334
Blood volume (mL/100 mL)	8.5 ± 3.9	7.01 ± 3.4	0.002
Permeability (mL/100 mL/min)	16.5 ± 8.2	15.1 ± 9.0	0.353
Mean transit time (s)	6.9 ± 3.5	5.9 ± 2.4	0.027
RECIST-1.1 diameter (mm)	58.2 ± 23.1	47.0 ± 21.4	< 0.000
Tumour volume (cc)	107.1 ± 138.5	54.6 ± 83.6	< 0.000

Data are given as means ± standard deviations with ranges in parentheses. *RECIST* Response evaluation criteria in solid tumours

**Fig. 2:**
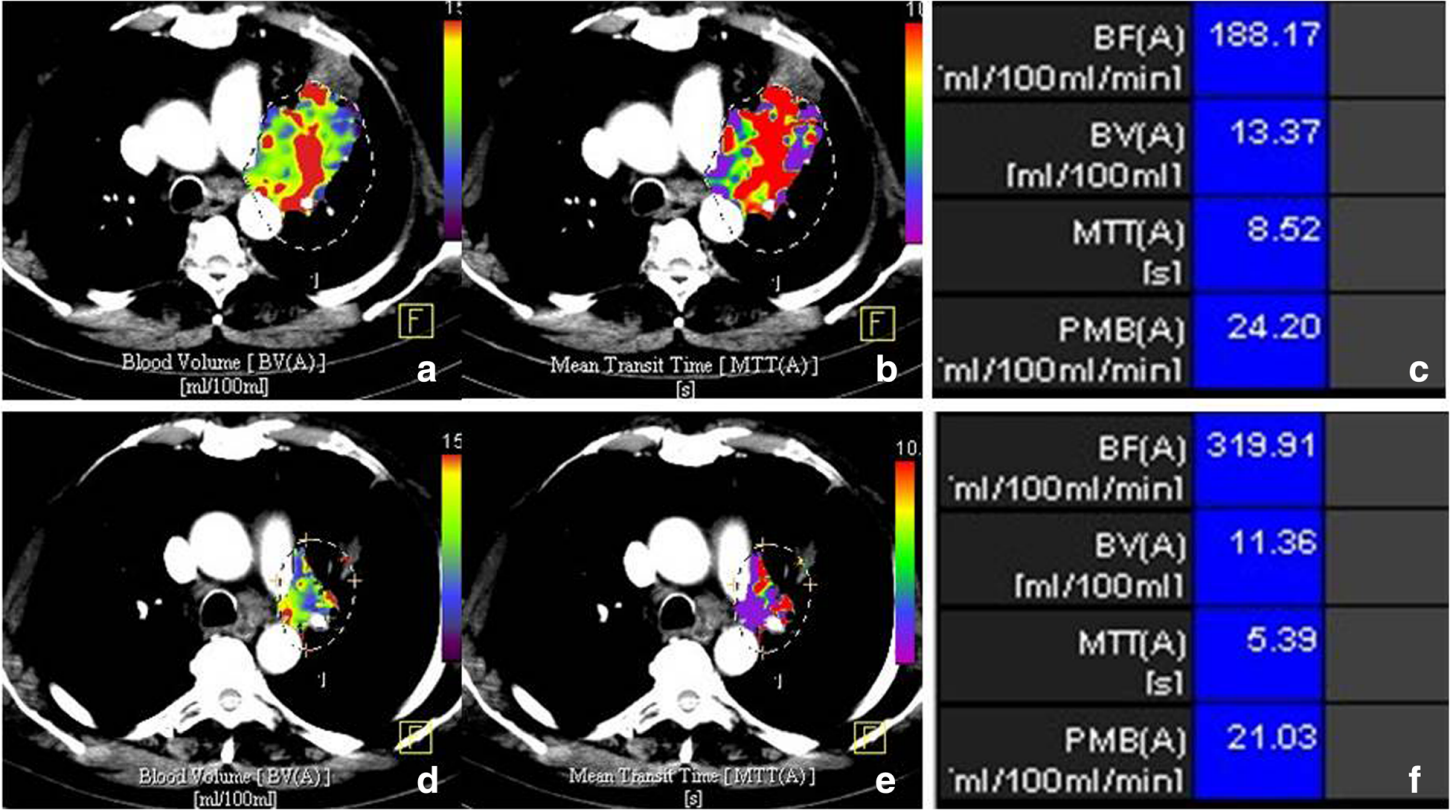
Perfusion computed tomography of left upper lobe epidermoid carcinoma. Colour coded maps and quantitative values of blood volume and mean transit time before (**a**, **b**, **c**) and after (**d**, **e**, **f**) platinum derivatives treatment show a decrease in these two perfusion parameters after treatment. *BF* Blood flow, *BV* Blood volume, *MTT* Mean transit time, *PMB* Permeability

Table 3 shows the changes in the perfusion parameters as a function of the three levels of treatment response according to RECIST-1.1. The patients with PR had a significant decrease of 21% in BV and of 17% in MTT. The PMB decreased by 10% and BF decreased by 5% (Fig. 2). A non-significant decrease in all perfusion parameters was found in the patients with SD: 15% for BV, 9% for MTT, and 17% for BF; PMB showed practically no variation. The three patients who presented PD had adenocarcinomas. The MTT decreased by 10%, and BV, BF, and PMB did not vary (*p* > 0.809).

**Table 3:** Changes in perfusion parameters, RECIST-1.1 diameter, and tumour volume in patients with partial response, stable disease, and progressive disease (*n* = 53)

	Partial response (*n* = 30)			Stable disease (*n* = 20)			Progressive disease (*n* = 3)		
	Baseline	Control	*p* value	Baseline	Control	*p* value	Baseline	Control	*p* value
Blood flow (mL/100 mL/min)	162.0 ± 78.8	154.7 ± 90.2	0.681	234.0 ± 199.0	195.3 ± 111.8	0.391	171.7 ± 146.2	170.41 ± 130.3	0.968
Blood volume (mL/100 mL)	8.1 ± 3.1	6.4 ± 3.0	**0.006**	9.3 ± 4.9	7.9 ± 3.7	0.137	7.3 ± 2.8	7.3 ± 4.8	0.975
Permeability (mL/100 mL/min)	15.5 ± 5.7	13.9 ± 8.3	0.307	17.8 ± 11.3	16.5 ± 10.2	0.709	18.3 ± 6.5	17.7 ± 9.6	0.809
Mean transit time (s)	7.1 ± 3.1	5.8 ± 2.4	**0.031**	6.4 ± 4.1	5.8 ± 2.3	0.456	7.8 ± 3.5	7.0 ± 3.6	0.465
RECIST-1.1 diameter (mm)	59.8 ± 23.7	41.0 ± 18.0	**0.000**	55.7 ± 23.4	53.3 ± 23.2	0.007	58.7 ± 20.4	65.0 ± 26.2	0.210
Tumour volume (cc)	120.2 ± 154.0	44.6 ± 59.8	**0.000**	90.4 ± 122.3	64.3 ± 108.0	**0.006**	93.3 ± 73.4	126.7 ± 97.6	0.145

Significant *p* values are in bold

*RECIST* Response evaluation criteria in solid tumours

Patients who received concomitant treatment with RT presented PR in the first PCT control, and a non-significant decrease was found in all of the perfusion parameters.

No significant correlation was found between the changes in the size and volume of the tumour and the changes in the values of any of the perfusion parameters evaluated in this study (*p* ≥ 0.220).

The most frequent histological types were adenocarcinomas (*n* = 28) and epidermoid carcinomas (*n* = 16). There were no significant differences in perfusion parameters in the baseline study between adenocarcinomas and epidermoid carcinomas: BF 185.73 *versus* 198.41, *p* = 0.752; BV 7.98 *versus* 7.77, *p* = 0.848; PMB 17.21 *versus* 16.14, *p* = 0.698; and TTM 6.66 *versus* 6.05, *p* = 0.560.

A non-significant decrease in all perfusion parameters in the control study compared to the baseline was observed for the adenocarcinomas (Table 4).

**Table 4:** Changes in perfusion parameters, RECIST-1.1 diameter, and tumour volume in adenocarcinomas (*n* = 28)

	Partial response (*n* = 16)		Stable disease (*n* = 9)			Progressive disease (*n* = 3)			
	Baseline	Control	*p* value	Baseline	Control	*p* value	Baseline	Control	*p* value
Blood flow (mL/100 mL/min)	157.7 ± 87.6	139.9 ± 85.0	0.467	240.3 ± 274.0	192.1 ± 115.8	0.639	171.7 ± 146.2	170.4 ± 130.3	0.968
Blood volume (mL/100 mL)	7.5 ± 3.3	6.8 ± 3.0	0.397	9.0 ± 4.9	7.9 ± 2.8	0.572	7.3 ± 2.8	7.3 ± 4.8	0.975
Permeability (mL/100 mL/min)	15.3 ± 5.6	15.1 ± 7.7	0.913	20.2 ± 13.9	16.6 ± 6.1	0.491	18.3 ± 6.5	17.7 ± 9.6	0.809
Mean transit time (s)	6.7 ± 3.25	6.4 ± 2.3	0.710	6.3 ± 1.6	5.8 ± 2.0	0.350	7.8 ± 3.5	7.0 ± 3.6	0.465
RECIST-1.1 diameter (mm)	58.1 ± 25.3	41.4 ± 19.3	**0.00**	54.2 ± 25.0	51 ± 24.92	0.041	58.7 ± 20.4	65.0 ± 26.2	0.210
Tumour volume (cc)	99.6 ± 163.9	35.7 ± 62.9	**0.051**	125.0 ± 168.4	96.6 ± 150.0	0.137	93.3 ± 73.4	126.7 ± 97.6	0.145

Significant *p* values are in bold

*RECIST* Response evaluation criteria in solid tumours

The changes in the BV values after the treatment were similar in the patients with PR and SD, showing a decrease by 9% and 12%, respectively, and were practically unchanged in the patients with PD. The MTT decreased by 4.6% in the patients with PR, 7.5% in the patients with SD, and 10.4% in the patients with PD. The PMB decreased by 20% in the patients with SD, 1.4% in the patients with PR, and 3% in the patients with PD.

The epidermoid carcinomas with PR presented a significant decrease by 32% in BV and by 30% in MTT. PMB decreased by 30%, approaching the statistical significance (*p* = 0.062). BF presented a non-significant increase of 5% (Table 5).

**Table 5:** Changes in perfusion parameters, RECIST-1.1 diameter, and tumour volume in epidermoid carcinomas (*n* = 16)

	Partial response (*n* = 10)			Stable disease (*n* = 6)		
	Baseline	Control	*p* value	Baseline	Control	*p* value
Blood flow (mL/100 mL/min)	176.1 ± 76.1	186.5 ± 106.4	0.775	234.9 ± 97.5	165.8 ± 108.7	0.010
Blood volume (mL/100 mL)	8.6 ± 3.3	5.9 ± 2.6	**0.007**	6.4 ± 2.8	5.2 ± 3.4	0.269
Permeability (mL/100 mL/min)	15.9 ± 6.9	11.1 ± 7.0	0.062	16.5 ± 11.5	6.7 ± 4.1	**0.036**
Mean transit time (s)	7.5 ± 3.6	5.3 ± 2.7	**0.018**	3.6 ± 1.7	5.3 ± 2.9	0.267
RECIST-1.1 diameter (mm)	54.5 ± 21.4	35.7 ± 15.7	**0.002**	62.5 ± 24.3	59.8 ± 23.9	0.082
Tumour volume (cc)	142.5 ± 171.8	56.1 ± 62.6	**0.038**	57.0 ± 4.6	22.5 ± 18.9	**0.021**

*RECIST* Response evaluation criteria in solid tumours

Significant *p* values are in bold

In the epidermoids with SD, a significant decrease was found in the BF and PMB values, which decreased by 29%and 59%, respectively, and BV decreased by 19% (*p* = 0.269). The MTT was the only parameter that increased after treatment, by 48%, although the increase was not significant (*p* = 0.267).

As for the remaining histological subtypes, of the five patients with undifferentiated carcinoma, two presented PR with a decrease in all perfusion parameters after treatment. Of the three patients with SD, one presented a decrease in BF and BV, an unchanged MTT, and an increase in PMB; one presented an increase in BF and PMB and a decrease in BV and MTT; and the third presented an increase in BF, BV, and PMB and a decrease in MTT.

Of the three patients with large cell carcinoma, two presented PR with an increase in PMB after treatment and a decrease in BV and MTT, and one presented an increase in BF whereas in another, this parameter decreased. The patient with SD presented an increase in BF, BV, and PMB and a slight decrease in MTT.

The only patient with a neuroendocrine tumour presented SD with a slight decline in BF, BV, and MTT, and an increase in PMB.

## Discussion

In our study, a decrease was observed in all perfusion parameters after treatment, which, in patients with PR, was significant for BV and MTT in epidermoid tumours, whereas no significant changes were observed in adenocarcinomas. This study suggests a limited capacity of the remaining perfusion parameters for determining the response to treatment. In patients with disease progression following the treatment, the changes were minimal in all NSCLC subtypes.

Because we used the RECIST-1.1 criteria, applying them only to the tumour lesion that was studied with PCT, without taking the remaining targeted lesions into account, it is possible that the changes in perfusion values were related to the change in tumour size. However, we did not detect a relationship between the percent variation of the perfusion parameters and the percent variation of the tumour diameter or volume, which is why these changes are attributable only to the variations in tumour vascularisation induced by treatment. This hypothesis is also supported by the fact that we did not observe changes in perfusion parameters in the baseline study between responders and non-responders [22]_._

The studies published to date in the literature show highly variable results, as summarised in Table 6. Wang et al. [23] also evaluated changes in the perfusion parameters induced by treatment with RT and CCT with platinum derivatives. However, there are two important differences with respect to our study: The first is that they did not evaluate the entire tumour but rather a size of 20 mm in the *z*-axis; therefore, in the majority of cases, there was no information about the entire tumour, which is a limitation, particularly when making evaluations before and after the treatment. The second is that they classified the patients into responders and non-responders, considering the tumour mass, which included different target lesions in various organs. Sudarski et al. [27] used the same protocol and did not found significant differences in any of the parameters in NSCLC but in the TTM in SCLC in a series of 100 patients treated with platinum derivatives.

**Table 6:** Comparison between the published studies and the current study

First author [reference]	Number of patients	Histology	CCT	RT	Targeted therapy	RECIST assessment	Blood flow	Blood volume	Mean transit time	Permeability
Wang et al [23]	35	Epidermoid Adenocarcinoma	No	Yes	No	Response	↓	↓	↑	↓ *p* > 0.05
Wang et al [23]	12	Epidermoid Adenocarcinoma	Yes	No	No	Response Progression	↕	↕	↕	↕
Fraioli et al [24]	45	Adenocarcinoma	Yes		Bevacizumab	Response		↓		↓
Lind et al [25]	23	Adenocarcinoma Epidermoid Large cell			Sorafenib Erlotinib	Response	↓			
Tacelli et al [26]	17	Adenocarcinoma	Yes		Bevacizumab	Response		↓	↓	
Tacelli et al [26]	23	Adenocarcinoma Epidermoid Large cell	Yes	No	No	Response		↔	↔	
Sudarski et al [27]	100	Adenocarcinoma Epidermoid	Yes	No	No	Response Progression Stable	↔	↔	↔	↔
Sudarski et al [27]	100	Small cell	Yes	No	No	Response Progression Stable	↔	↔	↓	↔
Trinidad et al [current study]	7	Adenocarcinoma Epidermoid Large cell	Yes	Yes	No	Response	↓ *p* > 0.05	↓ *p* > 0.05	↓ *p* > 0.05	↓ *p* > 0.05
Trinidad et al [current study]	46	Adenocarcinoma Epidermoid Large cell	Yes	No	No	Response		↓	↓	

Changes by treatment in perfusion parameters in lung cancer reported by different published studies:↑ = significant increase; ↓ = significant decrease; ↕ = in some patients, the parameter increases and in other patients, it decreases; ↔ = there were no changes; *p* > 0.05 nonsignificant changes

*RECIST* Response evaluation criteria in solid tumours

Fraioli et al. [24] found a significant decrease in BV and PMB in adenocarcinomas treated with platinum derivatives and bevacizumab. Therefore, their difference in the outcomes could be primarily attributable to the treatment with bevacizumab. PCT better reflects the changes secondary to antiangiogenic therapy, and bevacizumab has been shown to reduce perfusion values in colorectal cancer [28, 29]. Along the same line, Lind et al. [25] found a significant decline in BF in patients treated with the tyrosine kinase inhibitors sorafenib and erlotinib.

Tacelli et al. [26] concluded that CTP and, in particular, the BV and PMB parameters serve to evaluate the response to treatment with antiangiogenics but not the response to CCT. By contrast, we found a significant decrease in BV in the patients who responded to treatment with platinum derivatives when we analysed all of the histological subtypes and the epidermoid carcinomas, but the decrease in BV was not significant in the adenocarcinomas. In fact, this parameter varied the least at all response levels in this histologic subtype, although there were no differences in baseline perfusion parameters between adenocarcinomas and epidermoid carcinomas. Because the majority of the cases included in the study by Tacelli et al. [26] were adenocarcinomas, the discrepancy in the results may be explained by this difference.

Fraioli et al. [24] and Tacelli et al. [26] found a significant decline in PMB after treatment with antiangiogenics. PMB measures the flow of contrast from the intravascular to the extravascular space and is a reflection of the degree of immaturity of the tumour vessels, which are the main target of antiangiogenic therapy, and therefore, this type of therapy should decrease PMB. The effect of cytotoxic CCT, however, is a decrease in the generation of new vessels; thus, it has a lower impact on PMB and, instead, is expected to decrease parameters such as BF, BV, and MTT.

Fraioli et al. [24] highlighted a discrepancy between the RECIST-1.1 guidelines and the PCT approach, clearly reflected in the behaviour of the perfusion parameters in patients classified with SD. In the 28 adenocarcinomas evaluated in our study, the largest decline in all perfusion parameters occurred in the SD patients, with a significant decline in PMB in the epidermoids. These findings support the discrepancy between PCT and the RECIST-1.1 criteria. Even in the initial stages after treatment, PCT may reflect subtle changes in the biological activity of the tumour that are not translated into macroscopic changes in the tumour size; therefore, it may be more useful than the conventional size evaluated with RECIST-1.1.

There are studies of different lung cancer tumours that evaluated the changes in perfusion parameters following treatment with CCT and RT, with widely varying results [29–31]. These results suggest that PCT in lung cancer serves to monitor the response to treatment with targeted therapies, but its usefulness in treatment with cytotoxic RT and CCT is unclear. However, in our study the perfusion parameters, particularly BV and MTT, changed after these treatments and these changes appeared to be attributable solely to the treatment.

Taking into account that the majority of patients with advanced lung cancer receive platinum derivatives and RT as a first-line treatment protocol and the limitations of the response criteria based solely on tumour size (as are those of RECIST-1.1), PCT may have a role in monitoring treatment response. Despite the observed decrease in BV and MTT in patients with PR, larger studies are necessary to determine the usefulness of these parameters in predicting the degree of responsiveness and incorporating it into monitoring protocols, specifying what histologic subtype would most benefit from the quantitative analysis of the perfusion and defining the time intervals for control studies.

Our study has some limitations. The time elapsed between the baseline perfusion study and the initiation of treatment with CCT was variable. In patients with PR, the time elapsed between the initiation of treatment with CCT and the control perfusion study was significantly longer than in the non-responders. Although this finding could have affected the results, we believe that it was minimal because PCT was performed on the same day as the first standard computed tomography control and, therefore, the level of response reported in clinical practice did not vary. The same acquisition parameters and contrast dose were used, without taking the body mass index or tumour size into account, to meet the requirements of the kinetic model for calculating the perfusion values. Finally, we opted to apply the RECIST-1.1 criteria only to the tumour on which the perfusion study was performed because the perfusion values of the primary lesion cannot be extrapolated to the remaining lesions, and metastatic lesions may respond to the treatment differently than the primary tumour. Additionally, in the tumours treated with RT, this therapy only acts on the targeted lesion.

In conclusion, treatment with platinum derivatives, with or without RT, induces changes in PCT parameters. Except in the adenocarcinomas, PR is associated with a significant decrease in BV and MTT, attributable to the effect of the treatment on tumour vascularisation. Patients with no significant changes were obtained in any of the parameters.

## Abbreviations

BF: Blood flow
BV: Blood volume
CCT: Conventional chemotherapy
CR: Complete response
MTT: Mean transit time
NSCLC: Non-small cell lung cancer
PCT: Perfusion computed tomography
PD: Progressive disease
PMB: Permeability
PR: Partial response
RECIST: Response evaluation criteria in solid tumours
RT: Radiation therapy
SD: Stable disease
VPCT: Volume perfusion computed tomography
